# Commentary: A critical need for the concept of matrescence in perinatal psychiatry

**DOI:** 10.3389/fpsyt.2025.1601336

**Published:** 2025-05-30

**Authors:** Carolyn M. Friedhoff, Nancy Byatt, Sarah J. Palmer

**Affiliations:** ^1^ Lifeline for Families, Implementation Science and Practice Advances Research Center (iSPARC), Department of Psychiatry and Behavioral Sciences, UMass Chan Medical School, Shrewsbury, MA, United States; ^2^ UMass Memorial Health, Worcester, MA, United States

**Keywords:** matrescence, perinatal mental health, perinatal psychiatry, maternal mental health, community engaged research, qualitative research, parenthood, perinatal mortality

## 1 Introduction

We read Aurelie M. Athan’s article, “A critical need for the concept of matrescence in perinatal psychiatry.” (1) The concept of matrescence – developed by Dana Raphael in the 1970s – refers to the process of becoming a mother (2). Athan offers an updated conceptualization of matrescence, defining it as “a lifespan, developmental transformation that is biological, neurological, psychological, social, cultural, economic, political, moral, ecological, existential, and spiritual in nature.”^1(p3)^ Then, she calls for the integration of matrescence into the nosology of perinatal mental health disorders as part of a needed reform in maternal mental healthcare.

Integrating matrescence into perinatal psychiatry could help delineate pathological thoughts and behaviors in the perinatal period from expected challenges encountered in the transition to motherhood. It could also facilitate holistic maternal mental healthcare. Given that mental health and substance use disorders (SUD) are the leading underlying cause of pregnancy-related deaths in the United States (3), improvements in maternal mental healthcare are needed (4).

Although evidence-based treatments for mental health and SUDs exist, current US perinatal mental healthcare models are not meeting the needs or values of the individuals they aim to serve (5, 6). Perinatal individuals experience stigma when seeking and receiving mental healthcare and often feel dismissed by healthcare professionals (7). They want options for treatment beyond medications, including holistic patient-centered approaches to treatment and psychosocial interventions (7).

The integration of matrescence into perinatal psychiatry nosology would give attention to the psychosocial impact of the transition to motherhood. However, Athan’s plan does not offer a method to differentiate between expected challenges during matrescence and those that are indicative of psychiatric illness. In addition, the concept of matrescence is inherently gendered, thus limiting reform to benefit individuals who identify as women or mothers. Given the diverse identities of individuals who experience the transition to parenthood, a more inclusive concept than matrescence is needed to promote a complete and accurate understanding of mental health in parenthood. This commentary focuses on centering the lived experience of diverse matrescent individuals in psychiatry’s understanding of perinatal mental health. However, to address the United States’ perinatal mental health crisis, reform must also center the experiences of perinatal populations who do not identify as women.

## 2 Medicine’s exclusion of women’s voices

Women’s hermeneutical marginalization in research and clinical settings – wherein women have been excluded from contributing to academic medicine’s shared understanding of women’s mental health – hinders the field’s ability to address perinatal mental health concerns (8, 9). At a foundational level, medicine lacks understanding of mothers’ bodies because women have long been prohibited from participating in clinical trials (10, 11). Medicine’s understanding of perinatal mental health is further delayed by the stigmatization and criminalization of perinatal mental health challenges (12). Clinical assessments of perinatal mental health and SUDs are based on the information that patients and their supports disclose. However, the perinatal individuals in greatest need of mental healthcare are the least likely to disclose mental health concerns (13). Perinatal individuals hesitate to disclose mental health concerns because they fear compulsory psychiatric hospitalization, stigma associated with mental illness diagnoses, and loss of parental rights (7).

## 3 A plan for change

Athan’s suggested approach to reforming maternal mental health nosology envisions more “comprehensive and compassionate” healthcare services.^1(p2)^ But since it does not specify the methodology for progress, it is vulnerable to mental health stigma, racism, sexism, and other prejudices that have shaped medical research and practice. One might ask, “*For whom* will the reformed services be more comprehensive and compassionate?”

For example, Athan aims to “[establish] foundational knowledge of the unique developmental tasks and coping mechanisms of matrescence.”^1(p5)^ Yet over four decades of research have produced numerous scales comparing matrescence to maternal mental health challenges (14). All the while, many scales have not been validated outside of white female populations (14). Expectations around parenthood vary between different cultures (15–17). What are seen as normal thoughts and behaviors around the transition to motherhood in one culture may be seen as pathological in another. To produce scales and definitions that serve diverse perinatal individuals, studies of matrescence must center the insights of diverse populations with lived experience of mental health challenges.

## 4 Partnering with individuals with lived experience to develop equitable maternal mental health care

Researchers face challenges in recruiting diverse populations to participate in studies because of mistrust stemming from the abuse of Black, Latine, LGBTQIA2S+, and other marginalized populations in research (18). However, groups that have been marginalized face critical challenges in perinatal mental healthcare, such as access to care and higher mortality rates (19). For care to be accessible and neither overmedicalize nor dismiss their concerns, any reforms to the field must center the lived experience of marginalized groups.

To address the bias vulnerability in Athan’s proposed plan, we propose a methodology of community-engaged qualitative research. Community-engaged research is “a process of inclusive participation that supports mutual respect of values, strategies, and actions for authentic partnership” between researchers and community members (20). It works to increase trust in medical research and produce more acceptable interventions as community members collaborate with researchers to shape studies from design to dissemination (21).

To systematically analyze and incorporate the experiences of diverse individuals with lived experience in perinatal mental healthcare interventions and delivery, we propose qualitative research methods. Qualitative research centers the perspectives of study participants by eliciting first-hand accounts of their lived experiences. Qualitative analyses allow researchers to understand trends in experiences which inform implementation strategies and new directions for research. Community-engaged qualitative research allows lived experience to shape research goals and center the needs of communities that academic medicine aims to serve in reform.

## 5 Discussion

Community-engaged qualitative research could strengthen Athan’s proposal to reform US maternal mental healthcare. By centering the perspectives of individuals and communities who have been excluded from academic medicine’s efforts to define maternal mental health, this strategy can help academic medicine understand mental health among individuals from diverse cultures and identities. Reform efforts must center the voices of those with lived experience such that US medicine can adopt a more inclusive and patient-centered understanding of the transition to parenthood and address the perinatal mortality crisis.

## References

[1] Athan AM. A critical need for the concept of matrescence in perinatal psychiatry. Front Psychiatry. (2024) 15:1364845. doi: 10.3389/fpsyt.2024.1364845 38962063 PMC11220490

[2] Matrescence RD. Becoming a mother, a "new/old" rite de passage. In: Being female: reproduction, power, and change, 1st ed. [monograph on the Internet]. The Hague: Mouton Publishers; (1975). Available from: https://play.google.com/books/reader?id=84hyfRRHeakC&pg=GBS.PP1&hl=en p. 65–71 (Accessed May 13, 2025).

[3] Trost SL Beauregard J Chandra G Njie F Berry J Harvey A. Pregnancy-related deaths: data from maternal mortality review committees in 36 US states, 2017–2019. Education [Internet]. (2022) 45(10):1–0. Available from: https://champsonline.org/assets/files/Resources/ClinicalDocs/DiseaseCondition/MaternalMortalityReviewCommitteePregnancyRelatedDeathsData2017-2019.pdf (Accessed May 13, 2025).

[4] Wisner KL Murphy C Thomas MM. Prioritizing maternal mental health in addressing morbidity and mortality. JAMA Psychiatry. (2024) 81:521–6. doi: 10.1001/jamapsychiatry.2023.5648 38381408

[5] Cherkin DC Deyo RA Sherman KJ Hart LG Street JH Hrbek A. Characteristics of visits to licensed acupuncturists, chiropractors, massage therapists, and naturopathic physicians. J Am Board Fam Med. (2002) 15:463–72. Available from: https://www.jabfm.org/content/jabfp/15/6/463.full.pdf (Accessed May 13, 2025). 12463292

[6] Tindle HA Davis RB Phillips RS Eisenberg DM. Trends in use of complementary and alternative medicine by US adults: 1997-2002. Altern Ther Health Med. (2005) 11:42. Available from: https://www.proquest.com/scholarly-journals/trends-use-complementary-alternative-medicine-us/docview/204833462/se-2 (Accessed May 13, 2025). 15712765

[7] Byatt N Simas TAM Lundquist RS Johnson JV Ziedonis DM. Strategies for improving perinatal depression treatment in North American outpatient obstetric settings. J Psychosom Obstet Gynaecol. (2012) 33:143–61. doi: 10.3109/0167482X.2012.728649 23194018

[8] Elliott GK Millard C Sabroe I. The utilization of cultural movements to overcome stigma in narrative of postnatal depression. Front Psychiatry. (2020) 11:532600. doi: 10.3389/fpsyt.2020.532600 33192649 PMC7661847

[9] Fricker M Peels R Blaauw M. Epistemic injustice and the preservation of ignorance Vol. 1. . Cambridge: Cambridge University Press (2016) p. 144–59.

[10] Holdcroft A. Gender bias in research: how does it affect evidence based medicine? J R Soc Med. (2007) 100:2–3. doi: 10.1177/014107680710000102 PMC1761670 17197669

[11] Liu KA Dipietro Mager NA. Women’s involvement in clinical trials: historical perspective and future implications. Pharm Pract (Granada). (2016) 14:0–0. doi: 10.18549/PharmPract.2016.01.708 PMC4800017 27011778

[12] Dopazo A. Mommy dearest?: postpartum psychosis, the american legal system, and the criminalization of mental illness. U Miami Race Soc Just Law Rev. (2021) 12:275. Available at: https://repository.law.miami.edu/umrsjlr/vol12/iss2/6 (Accessed May 13, 2025).

[13] Forder PM Rich J Harris S Chojenta C Reilly N Austin MP. Honesty and comfort levels in mothers when screened for perinatal depression and anxiety. Women Birth. (2020) 33:e142–50. doi: 10.1016/j.wombi.2019.04.001 31133524

[14] Maxwell D Leat S. Measuring becoming a mother: A scoping review of existing measures of matrescence. Best Pract Ment Health. (2023) 19:1–31. doi: 10.70256/497467quramm

[15] Valiquette-Tessier SC Gosselin J Young M Thomassin K. A literature review of cultural stereotypes associated with motherhood and fatherhood. Marriage Fam Rev. (2019) 55:299–329. doi: 10.1080/01494929.2018.1469567

[16] Frederick A Leyva K Lavin G. The double edge of legitimacy: How women with disabilities interpret good mothering. Soc Curr. (2019) 6:163–76. doi: 10.1177/2329496518797839

[17] Collins C. Is maternal guilt a cross-national experience? Qual Sociol. (2021) 44:1–29. doi: 10.1007/s11133-020-09451-2

[18] Ellard-Gray A Jeffrey NK Choubak M Crann SE. Finding the hidden participant: Solutions for recruiting hidden, hard-to-reach, and vulnerable populations. Int J Qual Methods. (2015) 14:1609406915621420. doi: 10.1177/1609406915621420

[19] Howell EA Mora PA Horowitz CR Leventhal H. Racial and ethnic differences in factors associated with early postpartum depressive symptoms. Obstet Gynecol. (2005) 105:1442–50. doi: 10.1097/01.AOG.0000164050.34126.37 PMC4302723 15932842

[20] Ahmed SM Palermo AG. Community engagement in research: frameworks for education and peer review. Am J Public Health. (2010) 100:1380–7. doi: 10.2105/AJPH.2009.178137 PMC2901283 20558798

[21] Holzer JK Ellis L Merritt MW. Why we need community engagement in medical research. J Invest Med. (2014) 62:851–5. doi: 10.1097/JIM.0000000000000097 PMC4108547 24979468

